# Optimization of Piezoresistive Response of Elastomeric Porous Structures Based on Carbon-Based Hybrid Fillers Created by Selective Laser Sintering

**DOI:** 10.3390/polym15224404

**Published:** 2023-11-14

**Authors:** Gennaro Rollo, Alfredo Ronca, Pierfrancesco Cerruti, Hesheng Xia, Emanuele Gruppioni, Marino Lavorgna

**Affiliations:** 1 Institute of Polymers, Composites and Biomaterials, National Research Council, Via Previati, 1, 23900 Lecco, Italy; alfredo.ronca@cnr.it (A.R.); pierfrancesco.cerruti@cnr.it (P.C.); 2 Institute of Polymers, Composites and Biomaterials, National Research Council Viale J.F. Kennedy, 80125 Naples, Italy; 3 Institute of Polymers, Composites and Biomaterials, National Research Council, Via Campi Flegrei, 34, 80078 Pozzuoli, Italy; 4 State Key Laboratory of Polymer Materials Engineering, Polymer Research Institute, Sichuan University, Chengdu 610065, China; xiahs@scu.edu.cn; 5 Istituto nazionale Assicurazione Infortuni sul Lavoro (INAIL), Centro Protesi, Via Rabuina, Vigorso di Budrio, 40054 Bologna, Italy; e.gruppioni@inail.it; 6 Institute of Polymers, Composites and Biomaterials, National Research Council, P. le Enrico Fermi, 80055 Portici, Italy

**Keywords:** selective laser sintering (SLS), thermoplastic polyurethane (TPU), multiwall carbon nanotubes (MWCNTs), graphene (GE), strain sensors

## Abstract

Recently, piezoresistive sensors made by 3D printing have gained considerable interest in the field of wearable electronics due to their ultralight nature, high compressibility, robustness, and excellent electromechanical properties. In this work, building on previous results on the Selective Laser Sintering (SLS) of porous systems based on thermoplastic polyurethane (TPU) and graphene (GE)/carbon nanotubes (MWCNT) as carbon conductive fillers, the effect of variables such as thickness, diameter, and porosity of 3D printed disks is thoroughly studied with the aim of optimizing their piezoresistive performance. The resulting system is a disk with a diameter of 13 mm and a thickness of 0.3 mm endowed with optimal reproducibility, sensitivity, and linearity of the electrical signal. Dynamic compressive strength tests conducted on the proposed 3D printed sensors reveal a linear piezoresistive response in the range of 0.1–2 N compressive load. In addition, the optimized system is characterized at a high load frequency (2 Hz), and the stability and sensitivity of the electrical signal are evaluated. Finally, an application test demonstrates the ability of this system to be used as a real-time wearable pressure sensor for applications in prosthetics, consumer products, and personalized health-monitoring systems.

## 1. Introduction

Flexible and lightweight strain sensors are critical components for applications such as touch panels [1], soft robotics [2], health monitoring [3], human motion detection [4], electronic skin [5], and wearable electronics [6]. Typically, polymer nanocomposite strain sensors exhibit piezoelectric [7], capacitive [8], or piezoresistive [9] transduction ability. In particular, piezoelectric and capacitive strain sensors face a challenge to achieve reliable measurements in conditions of static tension and/or weak strain, as in biomedical sensing applications [10], while piezoresistive sensors react to external forces/deformations with resistance changes. They can be designed by using piezoresistive material, electrodes, and insulating cover in order to protect the system from humidity and thermal stress [11]. Meanwhile, they provide high pressure sensitivity and quick responses without much energy consumption [12]. Polymer-based piezoresistive composite materials reinforced with copper [13], silver [14], and gold nanowires [15], metal particles [16], carbon-based fillers like carbon nanotubes (CNT) [17], graphene (GE) [18], and carbon black [19] have been reported. In these systems, the conductive filler is able to produce a variation of electrical conductibility when an external force/deformation is applied. In particular, carbon nanomaterials such as carbon nanotubes (CNTs) (including both single-walled carbon nanotubes (SWCNTs) and multi-walled carbon nanotubes (MWCNTs)), graphene, and carbon nanofibers are used for their intrinsic properties, like electrical conductivity, outstanding tensile strength, extraordinary stiffness, light weight, and impressive thermal resistance [20]. The mechanical stiffness of an individual MWCNT is around 250~300 GPa [21], showing thermal and electrical conductivities of 3000 Wm^−1^ K^−1^ [22] and 8 × 10^8^ S/cm [23], respectively. Therefore, when used as filler in polymeric composites, MWCNTs can simultaneously improve mechanical strength and toughness as well as the electrical and thermal conductivities [24]. Graphene, as a defect-free two-dimensional sheet of carbon atoms, exhibits remarkable characteristics such as extraordinarily high electron mobility, exceeding 2 × 10^6^ cm^2^ V^−1^ s^−1^ at room temperature, mechanical stiffness of 130 GPa, thermal conductivity of ~5000 Wm^−1^ K^−1^, and electrical conductivity of 10^8^ S/m [25,26].

Wearable strain sensors with high sensitivity, light weight, excellent flexibility, thin structure, and cost-efficiency are highly sought in healthcare and prosthetics applications [27], where the main goal is the creation of pressure sensors that can be customized in shape and size [28]. Traditional processing techniques such as injection molding or extrusion often fail to induce the formation of a percolative network of conductive filler due to the high shear forces. Compression melting works better but still has some limitations in terms of a conductive percolating network. In this scenario, the sensitivity of piezoresistive materials can be further improved by the creation of porous 3D structures, since bulk materials have a limited compression strain. However, a big challenge lies in obtaining piezoresistive composite porous structures with high sensitivity, strength, elasticity, and response rate as well as stability under high compression cycling. These properties not only depend on the material’s microstructure but also on the pore’s morphology and dimensions. The relatively recent 3D printing (3DP) technologies are able to reach a considerable degree of customization, thanks to the high freedom of design and wide range of processable materials. There are several 3DP technologies available on the market, the most used being Fusion Deposition Modelling (FDM), Stereolithography (SLA), and Selective Laser Sintering (SLS) [29]. Among them, SLS uses a high-power laser as the source of heat energy to sinter a thin layer of polymer powder (0.1–0.3 mm thick) on a defined region and bind them to form a three-dimensional solid structure [30]. Polyamides (PAs) such as PA11 and PA12 are the most used materials in SLS, and intensive research has been conducted on the development of multifunctional PA composites [31]. In recent years, other classes of polymers have been developed to fabricate flexible, smart, and wearable devices by SLS, such as polyethylene (PE), polyurethane (PU) [32], and poly(dimethylsiloxane) (PDMS) [33]. Thermoplastic polyurethanes (TPUs) are an excellent option for laser sintering applications due to their versatility and adjustable mechanical properties. For these reasons, TPUs are indicated for the development of wearable sensors, where a high degree of flexibility is required. In this regard, it has been recently demonstrated that the SLS manufacturing of TPU composites containing conductive nanofillers such as MWCNTs and GE represents a straightforward and efficient method for creating sensors with high strain sensitivity [34]. Recently, we reported the fabrication and characterization of piezoresistive cubic devices made with TPU and GE-MWCNTs (50/50) at a very low concentration (1 wt%) [35,36]. This composite material, named TPU/(GE-MWCNTs), was selected after a systematic study of the electrical, mechanical, and thermal properties of three printing geometries (diamond, gyroid, and Schwarz), obtained using triply periodic minimal surfaces (TPMS) and porous structures (i.e., 20, 40, and 60%) [35,36].

The aim of the present work is to investigate the correlation between the piezoresistive and morphological (overall dimension, porosity, and pore dimension) properties of the 3D-printed composite samples, with the final aim of miniaturizing the samples yet leaving their electromechanical properties unaltered. Starting from a diamond porous structure with 60% porosity, a streamlining process has been adopted to reduce both the diameter and the thickness of the composite samples as much as possible, leaving the quality of the electrical signal and the printability unaltered. Furthermore, the potential of these materials for applications in prosthetics, consumer products, and personalized health-monitoring systems has also been addressed by manufacturing a real-time wearable pressure sensor as a proof of concept. 

For the next generation of prosthetic devices and intelligent human–machine interfaces, the approach pursued in this study might further guide the development of ultralightweight, reproducible, and resilient wearable sensors.

## 2. Materials and Methods

A polyester-type thermoplastic polyurethane (TPU) powder was provided by Mophene3D CT90A (Nanjing, China). Graphene material was provided by The Sixth Element Materials (Changzhou, China). Multi-walled carbon nanotubes (MWCNTs-NANOCYL 7000) were purchased from NANOCYL (Sambreville, Belgium). Silica nanoparticles, mainly used to promote the flow of TPU particles, consist of fine powder with a particle size of less than 10 nm, and it was purchased from Nanjing Tianxing New Material Co., Ltd. (Nanjing, China). All the materials and reagents were used as received.

The preparation of composite powder compatible with Selective Laser Sintering (SLS) plays a crucial role in determining the distribution of nanofillers within the polymer matrix. This, in turn, affects the structural and functional properties of the SLS-printed structures. In this study, the MWCNTs and graphene were used in a weight ratio of 50/50. The preparation method of the powder is described in a previous paper [37]. For comparison purposes, the control sample TPU/MWCNT composite powder (Mophene3D CT90A, Nanjing, China) was used as received without undergoing any additional preparation steps.

To design 3D porous structures, a mathematical approach has been proposed, using triply periodic minimal surfaces (TPMS) equations, as previously reported [35,36]. In this work, the attention has been focused on diamond (D) geometries with 60% (D60) and 20% (D20) porosity. The following trigonometric equations were used to design the porous D structures with the following boundary condition $\left(x^{2}+y^{2}\right)\leq {\left(6.0\cdot \pi \right)}^{2}\&\;\left|z\right|\leq 0.4\cdot \pi$

(1) $$\sin\left(x\right)\cdot \sin\left(y\right)\cdot \sin\left(z\right)+\sin\left(x\right)\cdot \cos\left(y\right)\cdot \cos\left(z\right)+\cos\left(x\right)\cdot \sin\left(y\right)\cdot \cos\left(z\right)+\cos\left(x\right)\cdot \cos\left(y\right)\cdot \sin\left(z\right)=C$$

**Figure 1:**
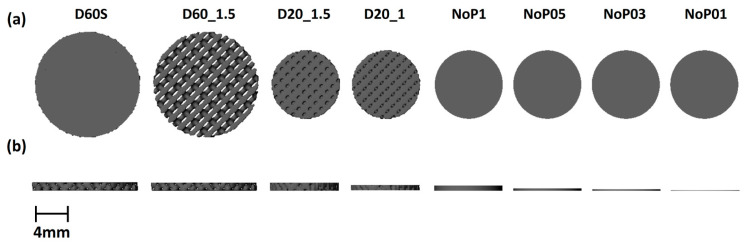
CAD visualization of the printed samples. (**a**) top view, and (**b**) lateral view.

Starting from our previous results to obtain a diamond structure with 20 and 60% porosity, the C value was set at 0.75 and −0.22, respectively [35], as previously reported. K3Dsurf v0.6.2 software (http://k3dsurf.sourceforge.net) was used to generate the D surface while the CAD files have been scaled to the required dimensions using Rhinoceros software 5.0 (Robert McNeel & Associates, Seattle, WA, USA). Different cylindrical non-porous (NoP) and porous (D) structures have been designed as reported in Figure 1, with and without two continuous layers (namely skin) and optimizing the electrical contact with electrodes. In order to evaluate the effect on the electrical properties of some variables (i.e., porosity, thickness, presence or absence of skins), the samples were designed and printed with the characteristics shown in Table 1. The CADs of the printed samples are shown in Figure 1. The starting point was a cylinder with diamond geometry and 60% porosity, measuring 20 × 2 mm^2^ with two continuous layers (namely skin—D60S), added to optimize the contact with the electrodes. A 20 × 1.5 mm^2^ (D60_1.5) disk was printed to evaluate the effect of the skins, maintaining the same porosity (the thickness of the sample goes from 2 mm to 1.5 mm just from elimination of the skins). Then, a cylinder measuring 13 × 1.5 mm^2^ with a porosity of 20% (D20_1.5) was printed to evaluate the effect of the miniaturization process on the electrical properties by reducing the thickness of the sensor but maintaining the same material volume. It was calculated that to reduce the diameter from 20 mm to 13 mm and maintain the same volume of materials, it was necessary to reduce the porosity from 60 to 20%, as reported in Table 1. The bulk volume (*V_b_*) is the overall volume of each sample without considering the inner porosity and it was calculated by the following equation:

(2) $$V_{b}=\pi \cdot r^{2}\cdot h$$

where *r* is the radius of the samples and *h* is the thickness. The real volume (*V_r_*) is the volume of the sample considering the porosity and it was calculated as follows:

(3) $$V_{r}=\left(1-\frac{P}{100}\right)\cdot V_{b}$$

where *P* is the sample porosity value.

To consider the effect of the sample thickness on the electrical properties, the D20 sample was reduced from 1.5 to 1 mm, obtaining D20_1. The final evaluations resulted in the removal of porosity without changing the thickness (NoP1), where NoP indicates that the structure is nonporous, resulting in a significant decrease in size within the values accepted by commercial electronic systems. Finally, the effect of the thickness on non-porous samples was evaluated by creating three different cylindrical prototypes with the following dimensions: 13 × 0.5 mm^2^ (NoP05), 13 × 0.3 mm^2^ (NoP03), and 13 × 0.1 mm^2^ (NoP01), as reported in Figure 1.

The D and NoP structures were 3D printed using the TPU/(GE-MWCNTs) composite powder by SLS (SnowWhite-Sharebot, Lecco, Italy). The device was equipped with a 14 W CO_2_ laser, which selectively fused the powder based on the stl model, using the process parameters reported in Table S1. To process the nanocomposite powder, the laser was set at 40% of the maximum energy and the laser speed to 40,000 points per second (pps).

After processing, to avoid part bending, the porous specimens were allowed to cool inside the equipment chamber until the powder bed temperature reached 50 °C, then they were recovered from the printer, and the excess powder was removed.

Scanning electron microscopy (SEM) observations were performed by a Fei Quanta 200 SEM (Hillsboro, OR, USA) to study the morphology of the porous structures. The samples were fixed on a support and metallized with a gold–palladium alloy to ensure better conductivity and prevent the formation of electrostatic charges.

To assess the compressive force sensitivity, reliability, and electrical signal stability, a Dynamic Mechanical Analysis instrument (DMA Q800, TA Instruments, New Castle, DE, USA) was employed in uniaxial testing setup. Figure S1 shows the setup employed for the electromechanical characterization experiment. The TPU/(GE-MWCNTs) porous systems were cyclically compressed in a quadratic loading–unloading test to apply compressive force in the range of 0.1 to 2 N. Simultaneously, to evaluate the load sensitivity and the repeatability, an Agilent 34401A 6½ Digit Multimeter (DM) was employed. The sample was inserted between two ceramic structures bearing two gold disks in the center. The connection to the multimeter was achieved using two copper conductive tape electrodes (Figure S1). The framework was placed in the DMA cells and connected to the DM by copper wires. The DM data acquisition software, specifically created by Labview 2016 software, continuously acquired resistance data. 

For the sample D20_1.5, a preload force of 0.1 N was necessary to ensure electrical conductivity. The electrical resistance at 0.1 N of preload was evaluated as R_0_. For all other samples, it was not necessary to impose any preload force. For all samples, the mechanical tests were performed in 20 cycles of 5 min each, at increasing loads of 0.1, 0.2, 0.5, and 1.0 N, at 25 °C. The electrical resistance of the specimen was monitored during the loading cycles. The strain sensitivity of the samples was expressed as Gauge Factor, GF = (ΔR/R_0_·ε), where ΔR/R_0_ is the resistance change rate and ε is the compression strain.

The sample with the best compromise in terms of sample miniaturization, stability, and reproducibility of the electrical signal was also characterized at high frequency (2 Hz) and minimum load (0.1 N) for 300 cycles to evaluate the electrical sensitivity at low loading. In addition, at the end of the characterizations at high loads (1 N), the drift effect of the material was evaluated. The drift effect is a variation in stability of a signal over time, typically after the application of pressure, measuring the time that occurs to allow a stable signal.

## 3. Results and Discussion

### 3.1. Design and Creation of the TPU/(GE-MWCNTs) Porous Structures

During the SLS process, the interface between TPU particles melts, allowing adjacent polymer particles to coalesce. Due to the high viscosity of the TPU, the near-zero shear stress of the melt flow during the laser sintering process enables the maintenance of the fillers at the interface between polymer particles as previously described [35,36]. In this study, TPU/(GE-MWCNTs) composite powder was used in SLS to build porous cylindrical samples with diamond geometries with two different porosities, 20% and 60%. Moreover, to further reduce the thickness from 1 mm to 0.1 mm, non-porous samples with cylindrical shapes have been printed. All the structures were successfully printed, except for the NoP01 sample, which showed some defects after printing due to the reduced thickness. The defects were mainly caused by the average dimension of the particles, which was in the same range of the sample thickness (≈100 µm), as reported in Figure S2.

### 3.2. Morphological Characterization of the SLS-Manufactured Porous Structures 

**Figure 2:**
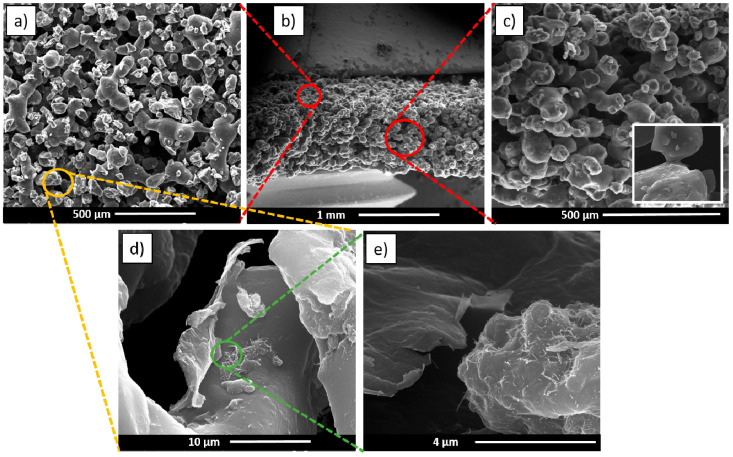
SEM observation of the NoP03 sample. (**a**) Low magnification of the surface; (**b**) lateral view of the fractured sample; (**c**) view along the thickness; (**d**,**e**) GE- and MWCNT-wrapped TPU particle surface at increasing magnification.

Figure 2 shows the SEM images of the NoP03 sample. The surface and the cryogenically fractured cross-section are visible in Figure 2a,c, respectively.

The effect of the sintering is clearly shown in Figure 2c, where is possible to note the polymeric neck connecting two adjacent particles. Increasing the magnification allows us to detect the presence of GE and MWCNTs on the surface of the particles (Figure 2d,e), which guarantees a synergetic effect on the electrical properties of the samples [36,38,39].

### 3.3. Electromechanical Characterization

**Figure 3:**
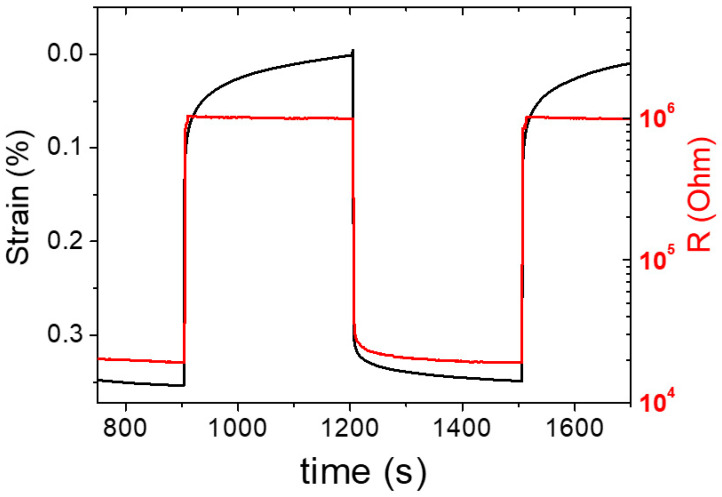
Load/unload cycles to demonstrate that the trend of R (red) follows that of the imparted deformation (black).

Figure 3 shows the mechanical deformation and the superposed electrical response of the 3D-printed samples. From the strain curve, a creep effect is noticed at lower strain, which indicates that the deformation of the material is not completely in phase with the applied load (which has a quadratic cycle). For the evaluation of the results, it should be considered that the materials have a typical viscoelastic behavior when subjected to load/unload cycles, and this can affect the deformation rate during the DMA test set in load control mode. Therefore, the material undergoes a slow deformation over time due the macromolecular relaxation. Consequently, R values were taken at the end of the load cycle.

It is worth noting that the R values follow the deformation during the loading step, but not during the unloading, where the electrical resistance reached the maximum value before the strain reached the peak. This behavior also highlights the stability of the signal at low loads (much lower than 0.1 N) of the composite. At such low loads, corresponding to strain values below 0.1%, no significant rearrangement of the percolation paths occurs that is able to produce a change in resistance.

In the first experiment, a quadratic loading–unloading test comprising loads from 0.1 to 2 N was conducted. Loading and unloading were held for one minute each. The resistance change response of the sensor to loading was continuously monitored.

**Figure 4:**
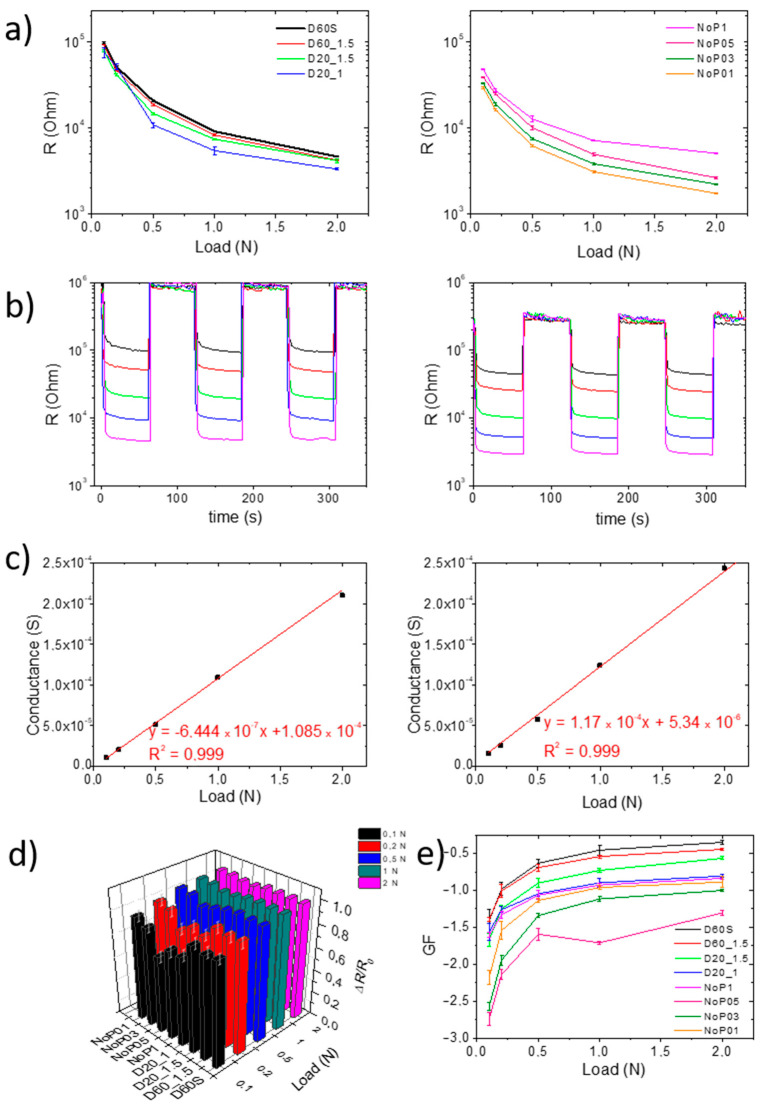
R values at relative load for (**a**) samples with D geometries and porosities from 20 to 60%, and samples without porosities; (**b**) electrical resistance variation at loads of 0.1 N (black), 0.2 (red), 0.5 (green), 1 N (blue), and 2 N (pink) of D60S (on the left) sample and NoP03 sample (on the right); (**c**) relative conversion into conductance to verify the linearity of the electrical response; (**d**) relative variation in electrical resistance at different loads for each sample; (**e**) Gauge Factor values for each sample as loads increase.

Only the first three cycles have been reported to show both the reproducibility of the data and the separation between two different load forces and two different cycles. In addition, a direct comparison between the samples D60S and NoP03 (which proved to be the best of the samples without porosity) is reported in this section. The other samples are shown in Figure S3. D60S showed excellent piezoresistive behavior, both in terms of ΔR and reproducibility, as noticed in Figure S3, where the R^2^ value of 0.999 demonstrates good linearity of the signal. This result demonstrates that the presence of the skins and the porous structure are a good compromise between the optimization of piezoelectric and mechanical behavior. Removing the skins (Figure S3, D60_1.5) also showed good results in terms of ΔR, reproducibility, and linearity of the electrical resistance signal with an identical R^2^ value of 0.999. This suggests that the skins have a marginal role in the electrical behavior. The effect of porosity going from 60 to 20% (Figure S3, D20_1.5) highlights a slight loss of linearity in the electrical signal, despite a slight decrease in the R^2^ value to 0.995. The reduction of the thickness to 1 mm (Figure S3, D20_1) brings about a decrease in the resistance values (Figure 4a, blue line), confirming the effect of this geometrical parameter on the electrical properties of the samples. Similarly, sample NoP1 shows a decrease in R values, with an R below 10–5 Ohm at 0.1 N (Figure 4a), but a nonlinear response when increasing the load to 1 N (Figure S3, NoP1) with an R^2^ value to 0.978.

By decreasing sample thickness, there is a consequent and significant decrease in the electrical resistance. As can be seen from Figure 4a, the NoP05 sample showed an excellent piezoresistive response to different loads, and high reproducibility, which was also confirmed by an R^2^ value of 0.990 (Figure S3, NoP05). The sample NoP03, in contrast, showed excellent repeatability and linearity of the electrical signal (R^2^ value of 0.999). Moreover, the range of the electrical resistance values is within the range usually found in commercial electronic systems. Figure 4b shows the comparison between the D60S and NoP03 samples at different load values, highlighting that the reduced thickness, combined with the absence of porosity, drastically reduced the electrical resistances. Notably, the comparison of the conductance underlines the linearity of the signals even for the 0.3 mm thin sample (Figure 4c). Figure S3 shows that NoP01 had good electrical behavior, while Figure S4 shows another specimen of the same sample that showed poor linearity and reproducibility in the signal. This low reproducibility is mainly due to the extremely reduced thickness of the NoP01 sample, which is comparable to the average dimensions of the powder microparticles (≈100µm), as reported in Figure S2. By calculating ΔR/R_0_ (Figure 4d), where ΔR is R_Load_ − R_0_ and R_0_ is the average value of electrical resistance at zero load for each sample characterized, and R_Load_ is the average value of electrical resistance at the set load (i.e., 0.1, 0.2, 0.5 N, etc.) for each sample, it is noted that the reproducibility of the signals does not depend on the size and geometry of the tested sample. The only exceptions to this trend are the D20_1.5 and NoP05 samples at 0.1 N load, and NoP05 at 0.2 N load, which showed values of ΔR slightly lower than the other samples. This difference may be ascribed to the higher material concentration for D20_1.5 and NoP05 samples, which results in good sensitivity. The effect is amplified by the small size of the samples and the small force used. 

The trend of the GF seen in Figure 4e can be explained by considering the sample structure. In fact, the lowest values (that is, the least negative ones, in this case) are ascribed to D60S and D60_1.5, which have the lowest density due to their high porosity. On the other hand, it is understandable that samples without porosity and with reduced thickness have higher absolute values of GF. In fact, results showed that GF increases as the thickness decreases. In any case, GF absolute values remain reasonably low if compared with larger samples [35], since the conductivity strictly depends on the distance traveled by the electrical current, which corresponds to the sample thickness. The comparison of the electromechanical performance of our elastomeric porous structures with similar composite systems in the literature indicates that the GF values are comparable, in particular for the non-porous samples. Indeed, Wang et al. [40] report a GF value of −2.6 for a CNT/epoxy system at a strain value very close to 0.1 N, while the GF values of our non-porous systems range from −2.1 to −2.8. Similarly, Lu et al. [41] studied a CNT/PDMS composite, and in this case, the GF values extrapolated for percentages of nanofiller comparable to those of the system under study are very similar.

**Figure 5:**
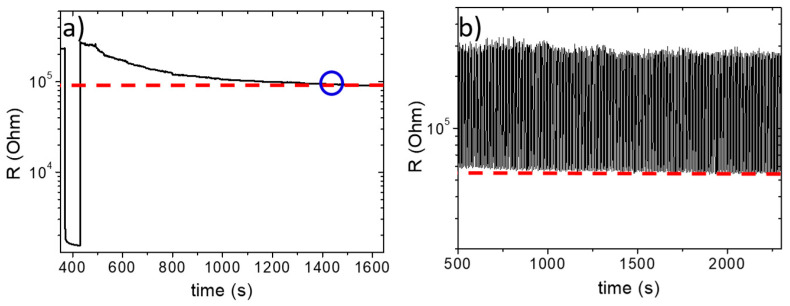
Electrical characterization of the NoP03 sample. (**a**) Drift effect evaluation, with indication of the time point where the plateau is obtained (blue ring); (**b**) high-speed response (2 Hz) at 0.1 N.

The best results in terms of the signal reproducibility, electrical resistance, GF trend, and quality of 3D printed samples are provided by the NoP03 sample. The drift effect, which is related to the time taken for the material and the correlated electrical signal to reach a plateau signal after having undergone a high load (2 N, in this case), was therefore evaluated for this sample (Figure 5a). For NoP03, the time to plateau is about 15 min and it allows the determination of a resistance value that can be set as a cutoff in electronic systems.

Furthermore, a test was performed to evaluate the electrical response to small and rapid load cycling (Figure 5b). A total of 300 cycles were performed at 2 Hz with a load of 0.1 N. It is seen from the graph that the sample responds instantly to the load imparted, always reaching comparable values of electrical resistance, confirming the reproducible behavior of the sample even at a load as low as 0.1 N.

### 3.4. Application Test

**Figure 6:**
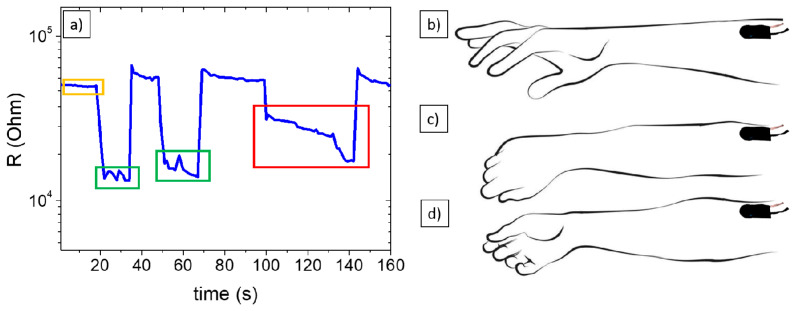
(**a**) Signal of electrical resistance to hand open (yellow, (**b**)), completely closed (green, (**c**)), and slowly closing (red, (**d**)).

Finally, to demonstrate the real applicability of such systems, the NoP03 sample was used to manufacture a strain/pressure device (as a proof of concept of a simplified sensor) able to monitor forearm movements. First, the piezoresistive material was connected to two copper electrodes and encased in an insulating cover with a layer of adhesive on one side (see Figure S5). This sensor was attached to a volunteer’s forearm and connected to a multimeter, then the signal was recorded when the hand was completely open (Figure 6b, signal indicated in yellow in Figure 6a).

Subsequently, the hand was closed completely (Figure 6c), twice. In Figure 6a, it is possible to see (green boxes) how the signal reaches similar values when the hand has been closed two times. The relative instability of the signal may be related to the high sensitivity of the piezoresistive material, capable of sensing even a small strain quickly, such as the natural muscular movement at the level of the forearm when closing the hand. To test this sensitivity, a third test was conducted, which consisted of closing the hand gradually and slowly (Figure 6d), and where the signal gradually decreased as the wrist was bent. From Figure 6a (red box), it is verified how the electrical resistance signal varies as the hand is closed and bent.

## 4. Conclusions

This study reports the miniaturization process of 3D-printed porous TPU/(GE-MWCNTs) piezoresistive sensors with a linear and repeatable response for smart wearable applications. In comparison with our previous work, the sensing materials developed in this work have been miniaturized, leaving the linear strain–resistance change response characteristics unaltered. Several samples were manufactured, starting from the large cylindrical sample D60S with a porous structure obtained by TMPS equation, to NoP01, which had a volume more than 20 times lower than D60S. The main goal of the miniaturization process was to reduce the overall dimension of the sample, optimizing piezoresistive properties and signal stability. Electromechanical strain–resistance characterization was conducted on all the printed samples to evaluate the repeatability of the electrical signal, its stability, and reproducibility as a function of morphological features such as porosity, size, and thickness. Non-porous samples having a diameter of 13 mm and a thickness of 0.3 mm exhibited a linear behavior for uniaxial compressive strains from 0.1 to 2 N, with associated Gauge Factor values of −2.6 and −0.99, respectively. These values are comparable to those reported in the literature for similar composite systems. The electromechanical characterization showed that the Nop03 sample, with an R^2^ value of 0.990, had resistance values readable by commercial electronic systems (between 10^4^ and 10^3^ Ohms), good linearity of the signal, and good reproducibility. Further characterizations were performed and the sensitivity to deformation at high frequencies (2 Hz) was evaluated, demonstrating an instantaneous response to mechanical stresses. The sensitivity of the proposed nanocomposite material was finally validated by manufacturing a sensor able to monitor hand position. Results suggest that the composite with the indicated formulation and dimensions can be used to develop a thin piezoresistive sensor with high strain sensitivity and potential applicability in human motion detection. Future work will be devoted to the thorough evaluation of the performance of the 3D-printed nanocomposite sensor, also addressing the influence of environmental factors such as temperature and humidity on its electromechanical properties. Furthermore, it will be interesting to design the materials with additional functionalities, such as antimicrobial activity, by formulating the SLS powder with antimicrobial additives.

## Figures and Tables

**Table 1:** Overview of sample dimensions in terms of diameter (d), height (h), bulk volume, and real volume.

Sample	Dimension (d × h) (mm·mm)	Porosity (%)	Bulk Volume (*V_b_*) (mm^3^)	Real Volume (*V_r_*) (mm^3^)
D60S	20 × 2	60	628.00	345.4
D60_1.5	20 × 1.5	60	471.00	188.40
D20_1.5	13 × 1.5	20	198.99	159.19
D20_1	13 × 1.0	20	132.66	106.13
NoP1	13 × 1.0	0	132.66	132.66
NoP05	13 × 0.5	0	66.33	66.33
NoP03	13 × 0.3	0	39.79	39.79
NoP01	13 × 0.1	0	13.26	13.26

## Data Availability

The data that support the findings of this study are available from the authors, upon request.

## Supplementary Materials

The following supporting information can be downloaded at: https://www.mdpi.com/article/10.3390/polym15224404/s1, Table S1: Process parameters; Figure S1: setup of characterization; Figure S2: Scanning electron microscopy (SEM); Figure S3: Electrical characterization; Figure S4: Sensor assembling. Figure S5: (a) Schematic representation of the sensors with piezoresistive composite (grey disc) sandwiched between copper electrodes (yellow) and protective cover (black), (b) a picture of the assembled prototype sensor.
