# Supplementary material for: Optimization of Piezoresistive Response of Elastomeric Porous Structures Based on Carbon-Based Hybrid Fillers Created by Selective Laser Sintering
Source: Polymers (Basel). 2023 Nov 14;15(22):4404. doi: 10.3390/polym15224404 (PMC10674563; doi:10.3390/polym15224404)
Supplement: Supplementary file 1 [file polymers-15-04404-s001.zip › polymers-2675769-supplementary.pdf]

Supporting information

Optimization of Piezoresistive Response of Elastomeric Porous Structures Based on Carbon-Based Hybrid Fillers Created by Selective Laser Sintering

Gennaro Rollo [1,*,†], Alfredo Ronca [1,2,†], Pierfrancesco Cerruti [1,3], Hesheng Xia [4], Emanuele Gruppioni [5] and Marino Lavorgna [1,6,*]

[1] Institute of Polymers, Composites and Biomaterials, National Research Council, Via Previati, 1, 23900 Lecco, Italy
[2] Institute of Polymers, Composites and Biomaterials, National Research Council Viale J.F. Kennedy, 80125 Naples, Italy
[3] Institute of Polymers, Composites and Biomaterials, National Research Council, Via Campi Flegrei, 34, 80078 Pozzuoli, Italy
[4] State Key Laboratory of Polymer Materials Engineering, Polymer Research Institute, Sichuan University, Chengdu 610065, China
[5] Istituto nazionale Assicurazione Infortuni sul Lavoro (INAIL), Centro Protesi, Via Rabuina, Vigorso di Budrio, 40054 Bologna, Italy
[6] Institute of Polymers, Composites and Biomaterials, National Research Council, P. le Enrico Fermi, 80055 Portici, Italy
* Correspondence: gennaro.rollo@ipcb.cnr.it (G.R.); marino.lavorgna@cnr.it (M.L.)
† These authors contributed equally to the work.

Process parameters

Table S1 shows the parameters used for the 3D printing of the samples.

Table S1. Sintering parameters adopted for processing TPU/(GE-MWCNTs) composite powders.

Process parameters	Value
Laser power (W)	14
Laser scan precision (μm)	100
Laser scan speed (pps)	40000
Environment temperature (°C)	90
Powder bed temperature (°C)	120
Outline laser power (W)	5,6
Layer thickness (μm)	100

Setup of characterization

Figure S1 shows schematically (Figure S1a) and by means of photos (Figure S1b,c) the setup used to characterize the composites, with a detail on the DMA used to impose cyclic loading/unloading and the multimeter used to collect the variations in the electrical resistance of the materials.

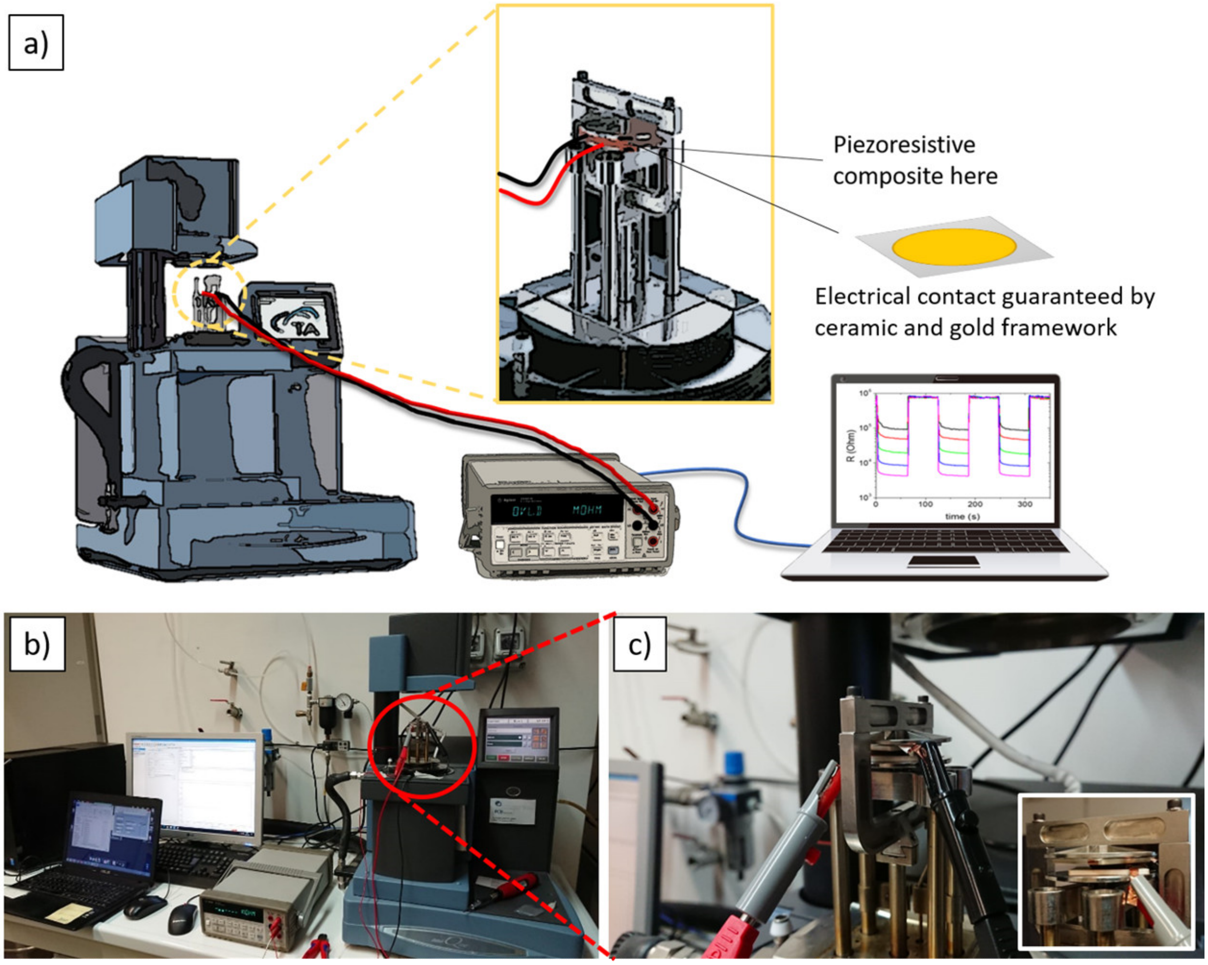

Figure S1. a) Schematic representation of the setup used for cyclic load/unload test, b) setup of piezoresistive properties measurement using the DMA coupled with multimeter, c) the DMA loading cell and the electrode connected to ceramic and gold plates with the tested sample.

Scanning electron microscopy (SEM)

Figure S2 of the SEM observation of the powders shows that the size of the powders ranges from 80 to 120 μm.

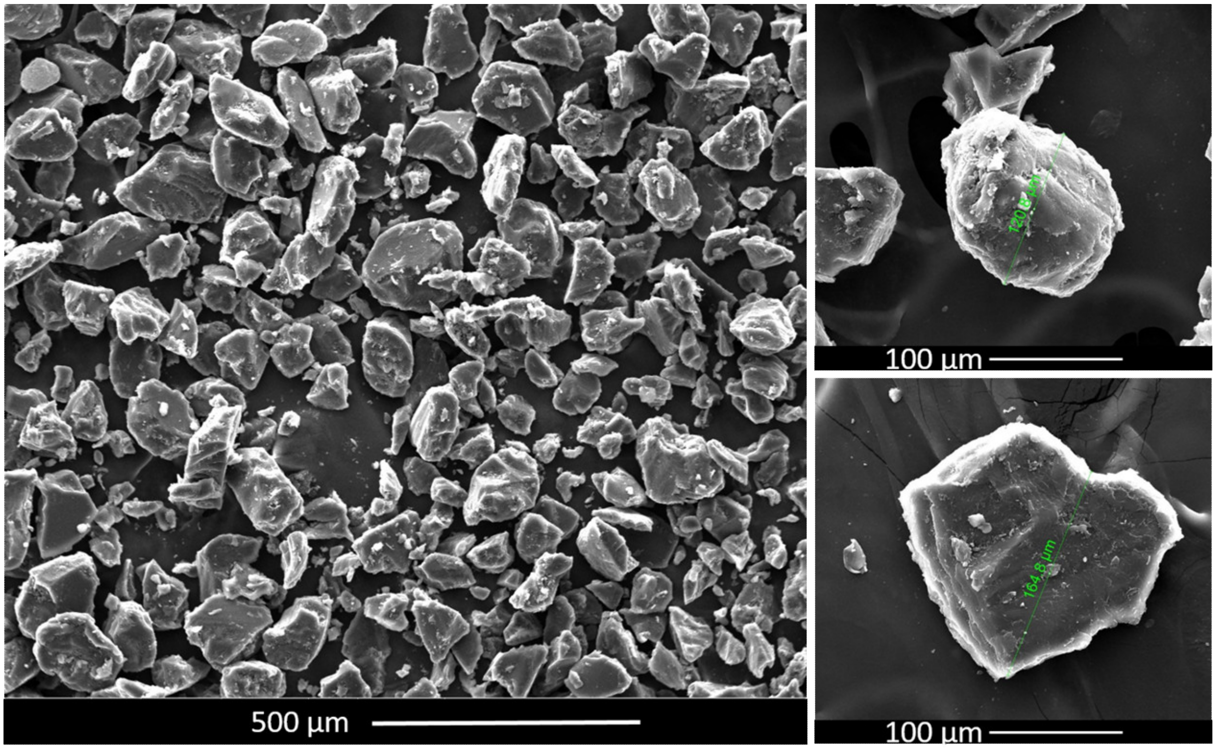

Figure S2. SEM of TPU/(GE-MWCNTs) (50/50) 1 wt% powder before sinterization.

Electrical characterization

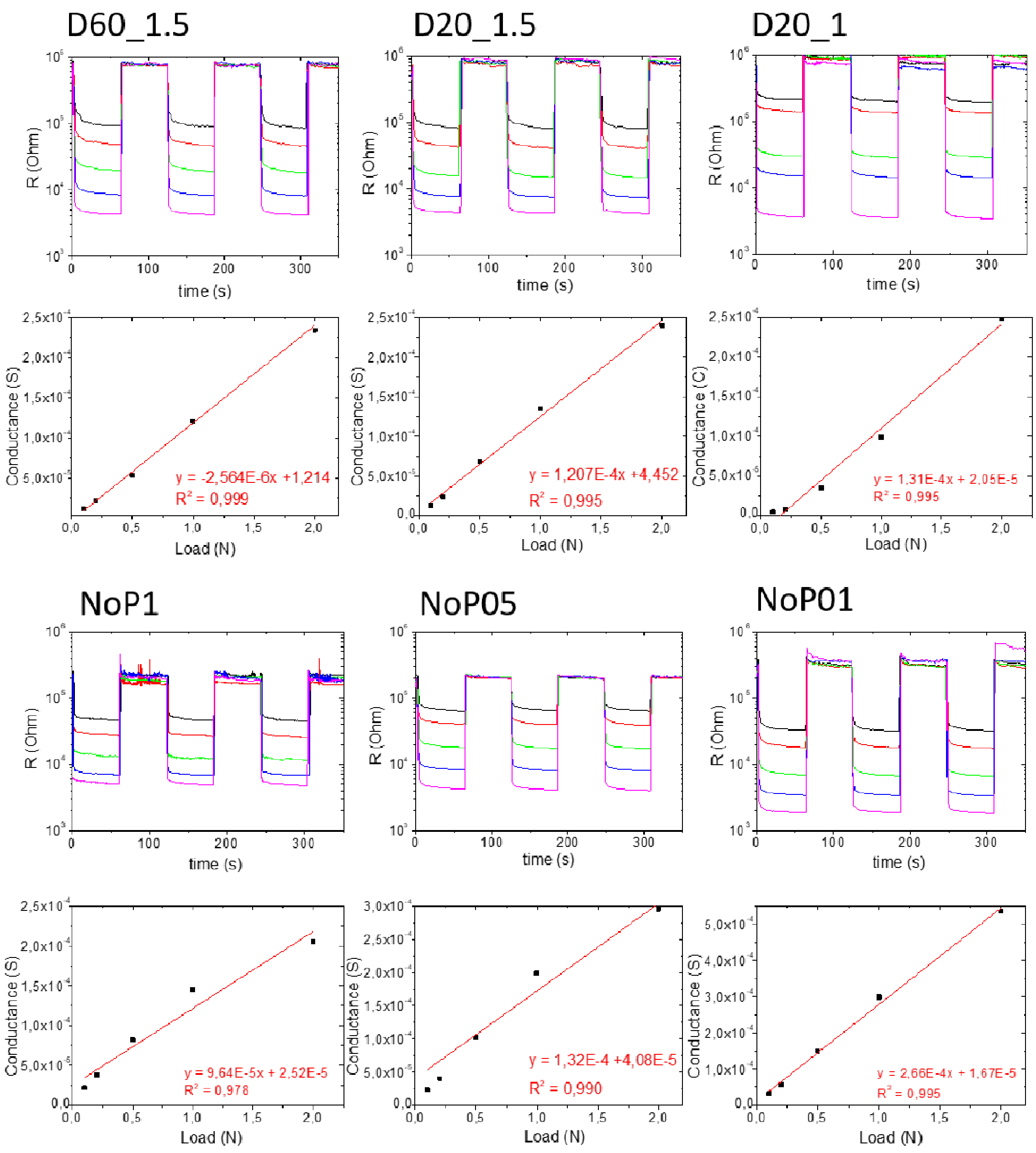

Figure S3. Electrical resistance variation at loads of 0.1 N (black), 0.2 (red), 0.5 (green), 1 N (blue), and 2 N (pink) of D60_1.5, D20_1.5, D20_1, NoP1, NoP05 and NoP01. Under each graph, the relative Conductance vs Load curves are reported, to verify the linearity of the electrical response.

Also due to the variable size of the powders, the NoP01 sample showed poor reproducibility both in the realization of the sample itself and in the piezoelectric characterization data. In Figure S4 it can be seen that NoP01 samples showed poor stability of the electrical signal and a non-linear response as a function of the load.

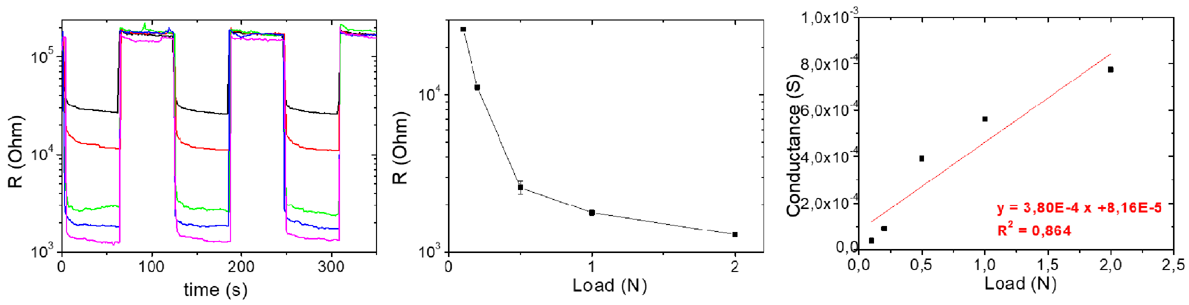

Figure S4. Electrical characterization of a specimen of NoP01

Sensor assembling

Figure S5 shows the simple assembly of the sensor system.

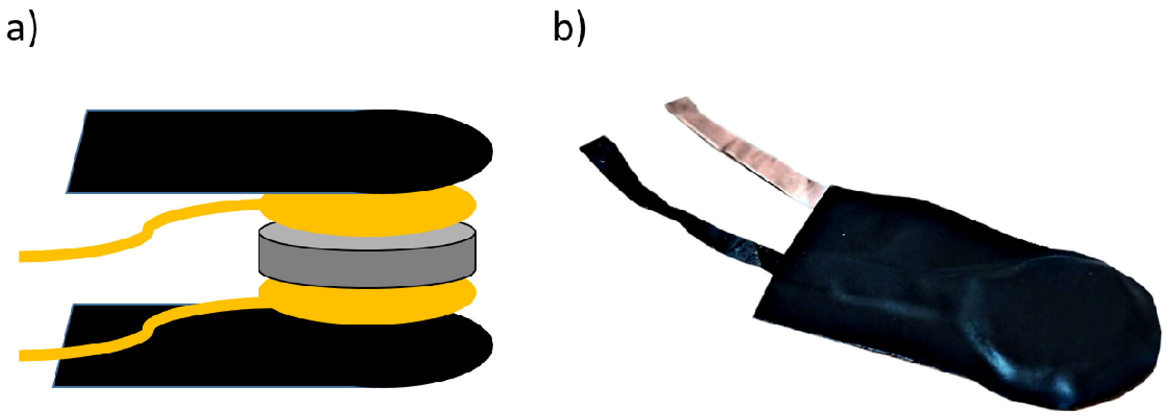

Figure S5. a) Schematic representation of the sensors with piezoresistive composite (grey disc) sandwiched between copper electrodes (yellow) and protective cover (black), b) a picture of the assembled prototype sensor.
